# From Identification to Enzymatic Activity Profiling: Comprehensive Characterization of BloCAβ, a Probiotic β‐Carbonic Anhydrase From *Bifidobacterium longum*

**DOI:** 10.1002/ardp.70333

**Published:** 2026-09-16

**Authors:** Simone Giovannuzzi, Andrea Ammara, Simone Carradori, Viviana De Luca, Marialucia Gallorini, Damiano Iacovozzi, Amelia Cataldi, Ilaria D'Agostino, Davide Moi, Valentina Onnis, Claudiu T. Supuran, Clemente Capasso

**Affiliations:** ^1^ NEUROFARBA Department, Section of Pharmaceutical Science University of Florence Florence Sesto Fiorentino Italy; ^2^ Department of Pharmacy “G. d'Annunzio” University of Chieti‐Pescara Chieti Italy; ^3^ Department of Biology, Agriculture and Food Sciences, CNR Institute of Biosciences and Bioresources Napoli Italy; ^4^ Department of Pharmacy University of Pisa Pisa Italy; ^5^ Department of Life and Environmental Sciences, Unit of Pharmaceutical, Pharmacological and Nutraceutical Sciences University of Cagliari, Cittadella Universitaria di Monserrato Monserrato Cagliari Italy

**Keywords:** activators, *Bifidobacterium longum*, carbonic anhydrase, HIEC‐6, macrophages, probiotic

## Abstract

The microbiota comprises communities of microorganisms that establish complex relationships with the host and play key roles in physiology, metabolism, and immunity. Increasing evidence highlights microbiota alterations in several diseases, particularly those driven by oxidative stress‐induced inflammation. Thus, identifying probiotic enzymes implicated in essential metabolic pathways is highly relevant to the development of future strategies targeting dysbiosis and related diseases. Herein, we report the first biochemical characterization of β‐class carbonic anhydrase (CA) from *Bifidobacterium longum* (BloCAβ) by inhibition and activation profiles. The enzyme was recombinantly expressed, purified, and kinetically characterized, displaying a catalytic efficiency within the same order of magnitude as, although lower than, that of human (h)CA I. The functional profile of this novel CA was explored using a panel of commercial sulfonamides, sulfamides, sulfamates, and inorganic/organic anions as inhibitors. Furthermore, several biogenic amines and amino acids were assessed as potential activators of BloCAβ, and their activation profiles were compared with those of hCAs I and II. The cellular effects of selected BloCAβ activators was further evaluated in human macrophages under basal and proinflammatory conditions, while the safety profile was assessed in human intestinal epithelial cells (HIECs). Among them, serotonin (**38**) was found to significantly enhance cell metabolic activity in both conditions and preserve the metabolic activity of HIECs, further supporting its biocompatibility. Overall, these results provide the first biochemical and functional characterization of BloCAβ and establish a biochemical basis for future studies investigating the physiological role of probiotic CAs in bacterial models.

## Graphical abstract

Production and biochemical characterization of a new β‐carbonic anhydrase from the probiotic *Bifidobacterium longum*, one of the main players of human gut microbiota.

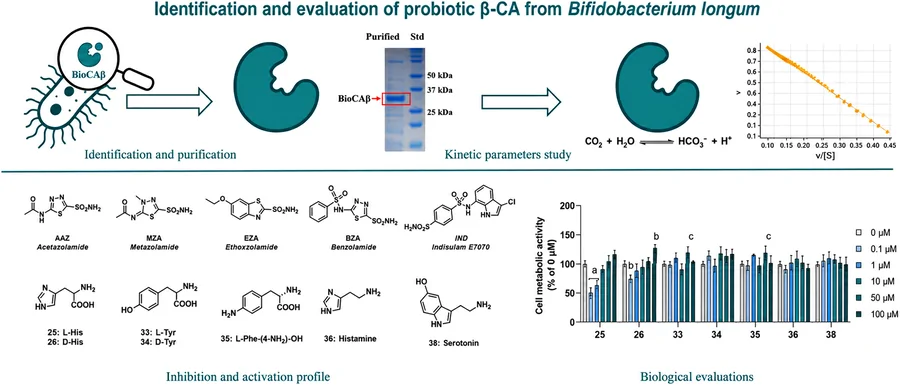

## 1 Introduction

Over the past decades, scientific and clinical paradigms have undergone substantial shifts, driven by advances in biotechnologies, chemoinformatics, and artificial intelligence (AI) that enable increasingly deeper insights into human physiology and disease [1]. Undoubtedly, advanced omics technologies have expanded our capacity to diagnose and treat pathological conditions through targeted precision‐medicine approaches. At the same time, modern healthcare is placing renewed emphasis on prevention, with growing efforts directed toward prophylactic strategies [2, 3]. This dual evolution in therapeutic and preventative medicine has also been strengthened by breakthroughs in high‐throughput sequencing, metagenomics, and computational biology, which allow rapid, cost‐effective, and highly accurate characterization of complex microbial ecosystems [4, 5, 6].

Such advances have catalyzed the emergence of a complementary domain to the long‐established field of vaccinology [7]: the study of the microbiota and the exploitation of microbiome‐directed therapeutics. The expansion of this field is supported by evidence that humans coexist with diverse microbial communities colonizing distinct anatomical niches, such as the gastrointestinal tract, vaginal mucosa, oral cavity, and skin, which collectively constitute the human microbiota [8, 9, 10]. These microbial populations perform essential metabolic, immunological, and regulatory functions. Notably, emerging data reveal intricate interactions between the microbiota and systemic physiology, with influences extending to the cardiovascular, endocrine, and nervous systems, including several gut axes [11, 12, 13, 14, 15, 16]. Under dysbiosis conditions, certain metabolites produced by gut bacteria have been linked to heightened levels of reactive oxygen species, which are a significant risk factor for neurodegeneration and cardiovascular diseases [17]. Given this complexity, scientific interest is increasingly focused not only on elucidating the taxonomic composition of microbial populations but also on understanding their functional capacities, interspecies interactions, and their ability to modulate host biological pathways, including oxidative stress‐induced inflammation. Within this context, probiotics, defined as live microorganisms that can confer health benefits to the host, have gained considerable interest [18, 19, 20, 21, 22, 23].

Mechanistic studies show that probiotics can restore microbial balance following dysbiosis, compete with pathogens for adhesion sites and nutrients, strengthen epithelial barrier integrity, produce antimicrobial metabolites, restore redox homeostasis, and modulate both innate and adaptive immune responses [24, 25, 26]. Clinical investigations are progressively evaluating probiotic species, such as *Lactobacillus* [27] *and Bifidobacterium* [28], for their therapeutic potential in gastrointestinal disorders, metabolic diseases, allergies, and even neuropsychiatric conditions [29, 30, 31, 32, 33]. Together, these advances describe a rapidly evolving field in which a deeper understanding of human microbiota is opening new possibilities for disease prevention and intervention, as well as innovative therapeutic strategies grounded in host‐microbe interactions.

In this dynamic scientific landscape, our research group, recognized for its long‐standing expertise in carbonic anhydrases (CAs; EC 4.2.1.1), is exploring the possibility of harnessing these enzymes for prophylactic purposes [34]. Briefly, CAs are ubiquitous metalloenzymes involved in a wide range of physio‐pathological processes, and although several isoforms have been extensively studied and their specific role proven, others remain under active investigation. CAs catalyze the reversible hydration of carbon dioxide (CO_2_) to bicarbonate anion (HCO_3_^−^) and a proton (H^+^), a fundamental reaction that underpins essential pathways, including pH homeostasis, carbon fixation, electrolyte balance, respiration, and buffering systems, among others. CAs are widely distributed in living organisms, with α‐class in humans (as in the physiologically relevant isoforms hCA I and hCA II), α‐, β‐, γ‐, and ι‐ classes in bacteria, and additional isoforms in fungi, protozoa, and plants [35].

Over the past 20 years, our group has evaluated structurally diverse natural compounds and generated extensive libraries of synthetic molecules as inhibitors of both human and microbial CAs, aimed at the identification of promising candidates for multiple biomedical applications, for example, anticancer, antiglaucoma, antibacterial, antiprotozoal, and antifungal treatments [35]. More recently, the activation of CAs emerged as a complementary research direction. In particular, several molecules, generally amino acids or amine‐based compounds, were found to be able to stabilize or facilitate the orientation of the proton‐shuttling residue, specifically His64 in hCA II, boosting the proton transfer more efficiently and enhancing the catalytic turnover of the enzyme, resulting in activating the enzyme [36].

In light of these findings and driven by the expanding interest in microbiome‐centered research, we sought to investigate the enzymatic properties and pharmacological modulation of probiotic CAs [34, 37]. Given that CAs play essential roles in the survival and metabolic adaptability of many microorganisms, we hypothesized that probiotic CAs could similarly act as key determinants of microbial fitness. Although this possibility is supported by the established physiological roles of CAs in microorganisms, direct evidence linking probiotic CA activation to enhanced bacterial fitness or probiotic functionality is still lacking. This hypothesis gains further relevance because dysbiosis is tightly associated with excessive production of ROS/RNS and redox‑sensitive inflammatory responses [38, 39]. Modulation of CA‑driven CO_2_/HCO_3_^−^ buffering could potentially affect probiotic metabolic adaptation; however, direct investigation in probiotic bacterial models is required. To test our hypothesis, we have selected relevant probiotic strains, including *Lacticaseibacillus rhamnosus* [40], *Limosilactobacillus reuteri* [37], and, herein for the first time, *Bifidobacterium longum*. In this work, we examined the genome of *B. longum* to identify genes encoding CA enzymes and, through recombinant DNA biotechnologies [41], we successfully expressed and isolated a β‐class CA, hereafter designated BloCAβ. We determined the kinetic parameters of BloCAβ and further characterized its functional profile by screening a broad panel of known sulfonamide‐ and anion‐based CA inhibitors, as well as a diverse set of amino acids and amines, to identify the most effective activators.

Our interest in *B. longum*, a Gram‐positive anaerobic bacterium belonging to the genus *Bifidobacterium*, stems from its well‐documented beneficial effects in humans [42, 43, 44, 45, 46]. Specifically, *B. longum* is among the most abundant microorganisms in the intestine of both infants and adults and has been reported to reduce the symptoms of colitis, relieve chronic inflammation, and contribute to the management of inflammatory bowel disease (IBD) [25] and lactose intolerance [47]. It has also been shown to play key roles in regulating the intestinal immune system and maintaining epithelial barrier function [48, 49, 50], although the underlying mechanisms remain only partially understood. Its medical relevance is further supported by the inclusion of several *B. longum* subspecies (*e.g*., *infantis*) and numerous strains in major probiotic databases, such as Probio‐ichnos (https://probio-ichnos.streamlit.app, accessed on 6 December 2025) [51], ProBioQuest (http://kwanlab.bio.cuhk.edu.hk/PBQ, accessed on 6 December 2025) [52], and PROBIO (https://bidd.group/probio/homepage.htm, accessed on 6 December 2025) [53], and several commercial probiotic formulations. Altogether, these features make this probiotic bacterium an ideal model for investigating the contribution of CA activity to probiotic efficacy. Finally, selected BloCAβ activators were evaluated for their effects on the metabolic activity of unstimulated and LPS‐stimulated human macrophages and for their biocompatibility in the HIEC‐6 intestinal epithelial cell line. These experiments were intended as preliminary in vitro evaluations in human cell models and did not directly assess BloCAβ‐dependent effects in bacteria.

## 2 Results and Discussion

### 2.1 Identification of a β‐class Carbonic Anhydrase From B. longum

**Figure 1:**
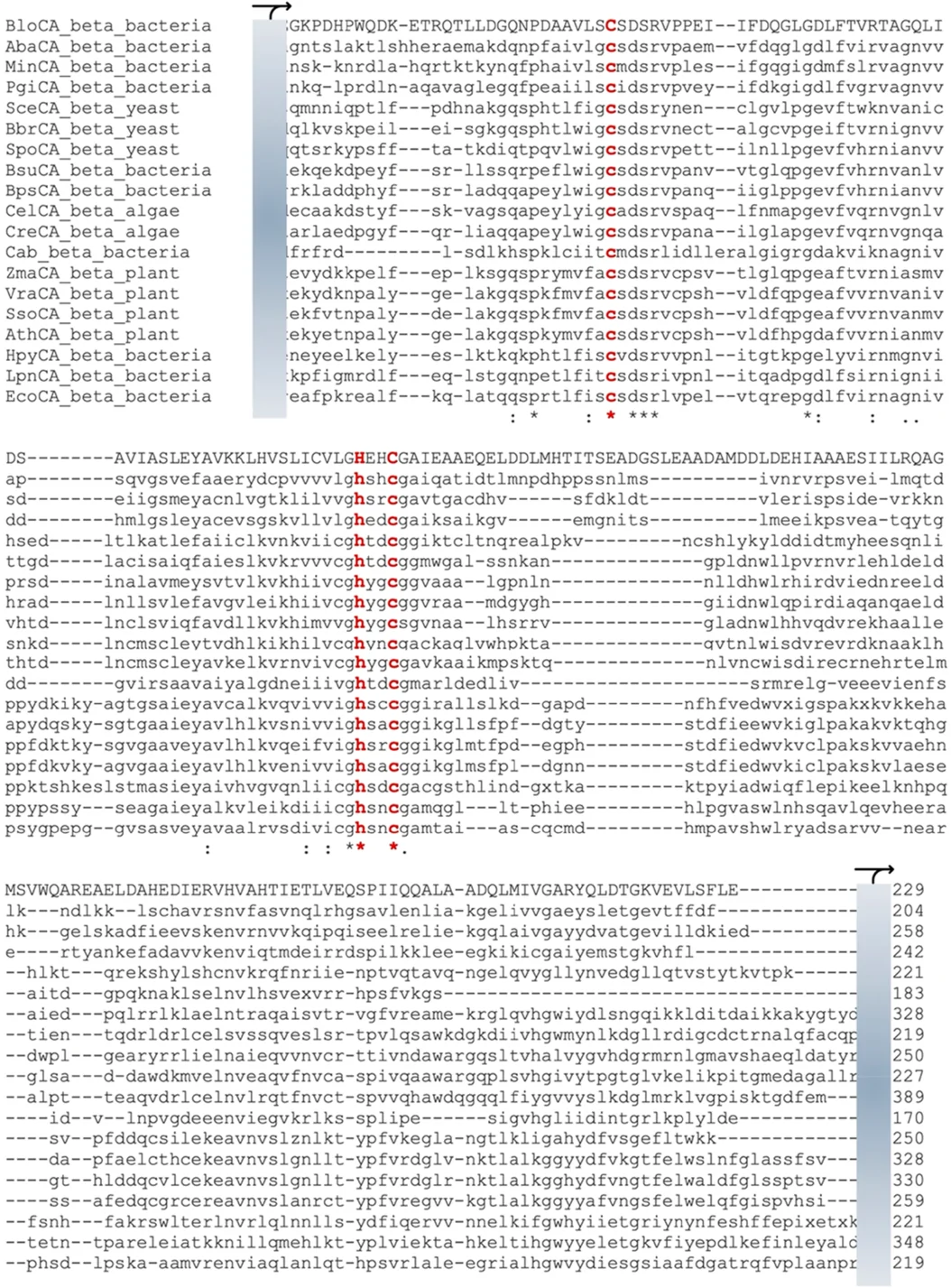
Multiple sequence alignment of BloCAβ with representative β‐CAs from bacteria, yeasts, algae, and plants. Amino acid sequences were aligned using Clustal Omega. For ease of visualization, only representative alignment regions containing conserved residues are shown. Arrows at the ends of the displayed alignment blocks indicate sequence continuation beyond the represented regions, whereas shaded triangles denote internal sequence segments omitted for graphical clarity. The conserved Cys–His–Cys triad involved in the coordination of the catalytic Zn^2+^ ion is highlighted in red. Asterisks (*) indicate fully conserved residues, colons (:) indicate conservation between residues with strongly similar physicochemical properties, and dots (.) indicate conservation between residues with weakly similar properties. Secondary‐structure elements are shown above the alignment, with arrows representing β‐strands and shaded boxes representing α‐helices. Numbers on the right indicate the total amino acid length of each sequence. Sequence identifiers and corresponding organisms are reported in Table 1.

A BLASTp search was performed using the β‐carbonic anhydrase from *Escherichia coli* (EcoCAβ; Accession No. WP_196083898.1) as a query to identify potential homologs in the *B. longum* genome. The analysis retrieved a single open reading frame encoding a 229‐amino acid protein (theoretical MW: 25094). This putative enzyme shared a significant sequence identity with β‐CAs from other bacteria, with *E*‐values < 1e^−30^. To verify this assignment, the amino acid sequence of this enzyme, BloCAβ, was aligned with representative β‐CAs from bacteria, yeasts, algae, and plants using Clustal Omega (Figure 1). The sequence acronyms and corresponding organisms are listed in Table 1.

**Table 1:** β‐Class CAs included in the multiple sequence alignment and phylogenetic analysis. For each enzyme, the acronym used, source organism species, taxonomic group, and protein accession number are reported.

Acronym	Species	Taxonomic group	Accession number
SsoCA	*Smallanthus sonchifolius*	Plants	KAI3713219.1
AthCA	*Arabidopsis thaliana*	Plants	NP_001330235.1
ZmaCA	*Zea mays*	Plants	ACG28885.1
VraCA	*Vigna radiata*	Plants	NP_001304227.1
AbaCA	*Acinetobacter baumannii*	Bacteria	WP_198920521.1
BsuCA	*Brucella suis*	Bacteria	WP_076772902.1
BpsCA	*Burkholderia pseudomallei*	Bacteria	WP_004550949.1
MinCA	*Myroides injenensis*	Bacteria	WP_010254382.1
LpnCA	*Legionella pneumophila*	Bacteria	WP_070321978.1
PgiCA	*Porphyromonas gingivalis*	Bacteria	WP_353307683.1
EcoCA	*Escherichia coli*	Bacteria	WP_196083898.1
Cab	*Methanothermobacter thermautotrophicus*	Bacteria	WP_048061095.1
HpyCA	*Helicobacter pylori*	Bacteria	WP_128026773.1
**BloCA**	***Bifidobacterium longum***	**Bacteria**	**WP_250234755.1**
CelCA	*Coccomyxia elongata*	Algae	CAL8465983.1
CinCA	*Chlamydomonas incerta*	Algae	KAG2427351.1
SpoCA	*Schizosaccharomyces pombe*	Yeasts	O94255.2
SceCA	*Saccharomyces cerevisiae*	Yeasts	NP_014362.3
DbrCA	*Brettanomyces bruxellensis*	Yeasts	VUG17928.1

Multiple sequence alignment revealed the presence of canonical zinc‐binding residues (Cys‐His‐Cys), which are characteristic of catalytically active β‐CAs. These conserved residues define the metal‐binding environment typical of β‐CAs and strongly support the classification of BloCAβ within this family. The sequence variations observed in the amino acid sequences are consistent with the natural divergence among bacterial homologs. To further assess the evolutionary relationships between BloCAβ and other β‐class CAs, aligned sequences were used to construct a phylogenetic tree. The resulting analysis revealed four major clades corresponding to bacterial, algal, plant, and yeast representatives (Supporting Information S1: Figure S1).

Within the bacterial group, BloCAβ clustered tightly with β‐CAs from *Acinetobacter baumannii* (AbaCAβ), *Porphyromonas gingivalis* (PgiCAβ), and *Myroides injenensis* (MinCAβ), as supported by high bootstrap values (> 90%). Other bacterial β‐CAs, such as those from *Escherichia coli*, *Helicobacter pylori*, and *Legionella pneumophila*, formed more distant clusters, whereas plant, algal, and yeast β‐CAs branched independently. The position of BloCAβ within this well‐supported bacterial subclade supports its annotation as a functional member of this enzyme family.

### 2.2 Recombinant Expression and Purification of BloCAβ

The gene encoding BloCAβ was cloned into an expression vector and heterologously expressed in *E. coli* Arctic Express (DE3) cells. The recombinant enzyme was produced with an *N*‐terminal extension of 37 amino acids, including a 6×His‐tag and linker sequence, which facilitated purification by immobilized metal affinity chromatography. After expression, BloCAβ was purified to homogeneity using nickel affinity chromatography.

SDS‐PAGE analysis (Supporting Information S1: Figure S2) revealed a major band with an apparent molecular mass of approximately 29 kDa, consistent with the expected size of the tagged recombinant protein, indicating successful purification of recombinant BloCAβ and an estimated purity of approximately 85%. The purified BloCAβ preparation was subsequently used for biochemical and kinetic characterization. The oligomeric state of recombinant BloCAβ was not investigated in the present study. Considering that β‐CAs frequently adopt oligomeric quaternary structures, future biophysical studies will be required to determine whether BloCAβ exists as a dimer, tetramer, or higher‐order assembly under physiological conditions.

### 2.3 Kinetic Parameters of BloCAβ

BloCAβ exhibits catalytic activity for the reversible hydration of CO_2_ into HCO_3_^−^ and proton (H^+^), showing a turnover rate (k_cat_ of 2.43‧10^5 ^s^−1^) higher than that of the hCA I (k_cat_ of 2.0‧10^5 ^s^−1^), but approximately 6‐fold lower than that of the highly efficient hCA II (k_cat_ of 1.4‧10^6 ^s^−1^, Table 2). Moreover, BloCAβ is less efficient than both human enzymes in terms of catalytic efficiency, *k*_cat_/*K*_M_ of 1.45‧10^7 ^M^−1^s^−1^, especially when compared with the highly efficient hCA II, *k*_cat_/*K*_M_ of 1.5‧10^8 ^M^−1^s^−1^. Many CAs rank as the most efficient natural catalysts, and BloCAβ still demonstrates significant activity, with a *K*_M_ of 16.8 mM. Such discrepancies in catalytic efficiency reflect underlying structural differences between the two enzyme classes of α‐ and β‐CAs. Such differences may influence the affinity and selectivity of inhibitors and activators, as will be further discussed. For instance, sulfonamide inhibitors, such as acetazolamide (AAZ), interact differently with those isozymes. This was particularly evident in the *K*_I_ values, reflecting the strength of these interactions. Indeed, AAZ exhibited a *K*_I_ of 250 and 12 nM on hCA I and hCA, respectively, while showing a *K*_I_ of 435 nM on BloCAβ, resulting in approximately 2‐ and 36‐fold more effective against the human isoforms, respectively. Consequently, we also investigated the sulfonamide inhibition profile of BloCAβ using substituted (hetero)aryl‐sulfonamides and clinically licensed drugs.

**Table 2:** Kinetic parameters for the CO_2_‐hydration reaction catalyzed by hCAs I and II and BloCAβ together with the corresponding K_I_ for AAZ [54].

Organism	Acronym	Activity level	*k*_cat_ (s^−1^)	k_cat_/K_M_ (M^−1^ s^−1^)	K_I_ of AAZ (nM)
*Homo sapiens*	hCA I^a^	Moderate	2.0 * 10^5^	5.0 * 10^7^	250
*Homo sapiens*	hCA II^a^	Very high	1.4 * 10^6^	1.5 * 10^8^	12
*B. longum*	BloCAβ	Moderate	2.43 * 10^5^	1.45 * 10^7^	435

*Note:* The catalytic turnover number (*k*_cat_), catalytic efficiency (*k*_cat_/K_M_), and inhibition constant (*K*_I_) of AAZ are reported. Data for hCA I and hCA II were obtained from Supuran, 2008 [55]. Enzymatic characterization was performed at 20°C under the following conditions: hCA I and hCA II, 10 mM HEPES buffer at pH 7.5; BloCAβ, 20 mM Tris buffer containing 10 mM NaClO_4_ at pH 8.3.

### 2.4 Enzymatic Activity Profiling of BloCAβ

To characterize the enzymatic profile of BloCAβ, a stopped‐flow CO_2_ hydration assay was performed using panels of recognized inhibitors and activators of CAs. No experimental 3D structure of BloCAβ is currently available in the Protein Data Bank (PDB, https://www.rcsb.org/). Although an AlphaFold‐predicted model has been released (https://alphafold.ebi.ac.uk/, AF^−^Q8G6M3‐F1), it represents the enzyme in a monomeric state. To further assess the feasibility of a computational approach, we performed a structural homology search against the PDB using the BLAST Tool. The analysis retrieved a top‐ranking hit with an extremely significant *E*‐value. However, despite this high statistical confidence in the fold recognition, the template shares only ~41% sequence identity with BloCAβ. While this level of conservation is widely accepted for fold prediction, reliable high‐throughput molecular docking requires a significantly higher degree of identity (> 50%) to ensure atomic‐level accuracy of side‐chain rotamers within the binding pocket. This is particularly critical for functional bacterial β‐CAs, which are obligate oligomers with the active site often located at the interface between subunits. Consequently, the monomeric AlphaFold model displays an artificially exposed active site, while reconstructing the correct quaternary interface from a moderate‐identity template introduces structural uncertainties that could generate artifacts in ligand‐binding simulations. Therefore, to adhere to a rigorous standard of structural interpretation, we prioritized the interpretation of inhibition/activation profiles based on the well‐established structural homology and conserved features of the β‐CA class rather than on potentially misleading molecular docking data and SAR rationalization.

#### 2.4.1 Inhibition Activity Profile of BloCAβ

**Figure 2:**
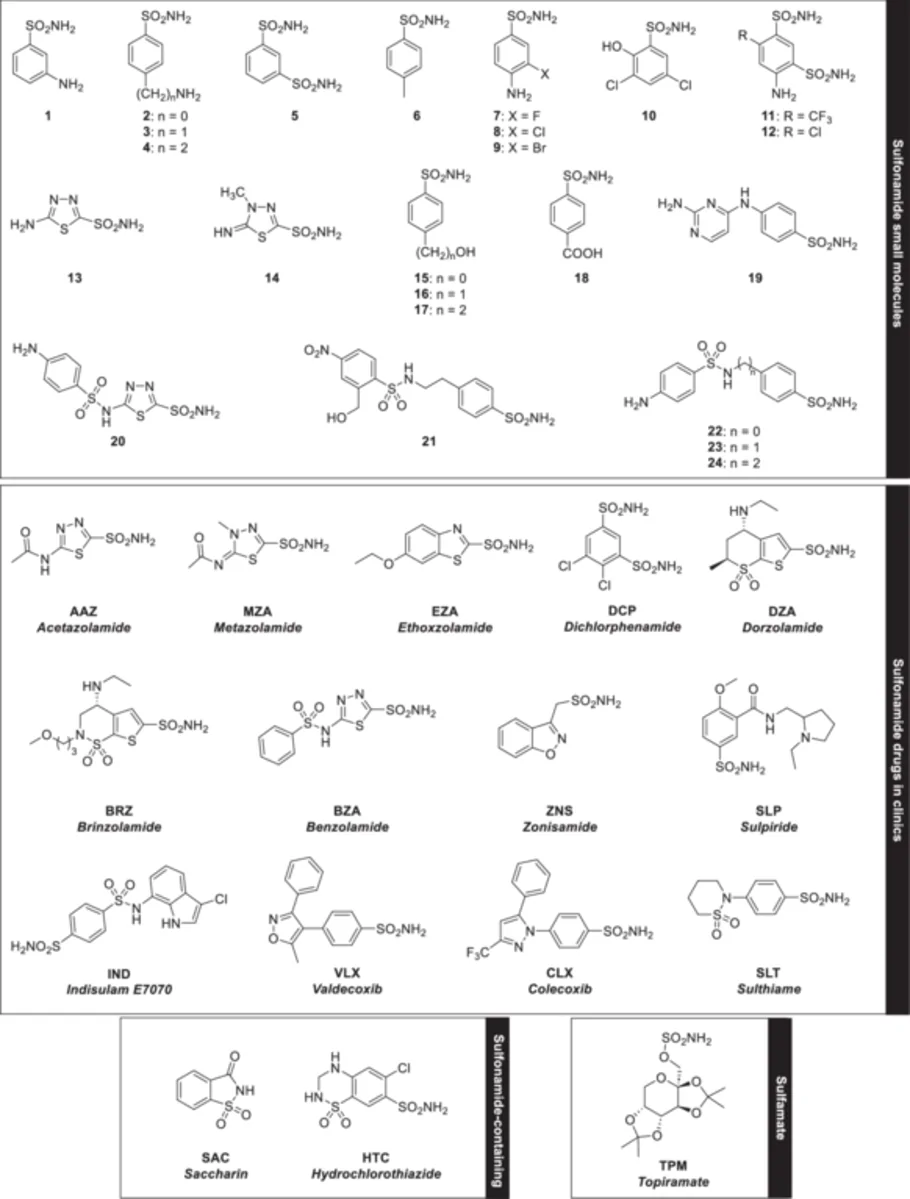
Chemical structures of the compounds used to assess the inhibition profile of BloCAβ. The 40 tested molecules are classified according to their main zinc‐binding functionality as primary sulfonamides, sulfonamide‐containing compounds, or sulfamates.

Among known CA inhibitors (CAIs), a library of 40 compounds (**1**–**24** and AAZ‐HCT) was selected, encompassing representative commercial simple aromatic and heterocyclic sulfonamides, sulfamates, sulfamides, and related compounds (Figure 2 and Table 3), along with (in)organic anions/compounds (Table 4). The inhibition constants (*K*_I_s) of these compounds against BloCAβ, together with comparative values for hCAs I and II, are reported in Tables 3 and 4.

**Table 3:** Inhibition of BloCAβ and hCAs I and II by sulfonamides and related compounds. *K*_I_ values were determined for compounds **1**–**24** and for the clinically used or reference compounds using a stopped‐flow CO_2_ hydration assay [54].

Cpd	*K*_I_ (nM)^a^		
	hCA I^b^	hCA II^b^	BloCAβ
**1**	28000	300	1177
**2**	25000	240	5091
**3**	79.0	8.0	8444
**4**	78500	320	9327
**5**	25000	170	795
**6**	21000	160	846
**7**	8300	60.0	5532
**8**	9800	110	7982
**9**	6500	40.0	12900
**10**	7300	54.0	24000
**11**	5800	63.0	17300
**12**	8400	75.0	20400
**13**	8600	60.0	569
**14**	9300	19.0	412
**15**	5500	80.0	711
**16**	9500	94.0	1493
**17**	21000	125	4333
**18**	164	46.0	4672
**19**	109	33.0	461
**20**	6.0	2.0	543
**21**	69.0	11.0	662
**22**	164	46.0	705
**23**	109	33.0	719
**24**	95.0	30.0	883
AAZ	250	12.0	435
MZA	50.0	14.0	281
EZA	25.0	8.0	89.4
DCP	1200	38.0	8269
DZA	50000	9.0	917
BRZ	45000	3.0	769
BZA	15.0	9.0	121
ZNS	56.0	35.0	1653
SLP	1200	40.0	19500
IND	31.0	15.0	485
VLX	54000	43.0	2533
CLX	50000	21.0	1955
SLT	374	9.0	1074
TPM	250	10.0	702
SAC	18540	5959	39300
HCT	328	290	23500

^a^ Mean of three independent determinations performed using the stopped‐flow CO_2_ hydration assay; experimental errors were within ±5%–10% of the reported values.

^b^ Data for hCA I and hCA II were taken from Bonardi et al., 2025 [37].

**Table 4:** Inhibition of BloCAβ and hCAs I and II by inorganic and organic anions and related compounds. *K*_I_ values were determined using a stopped‐flow CO_2_ hydration assay [54].

Anion/compound	*K*_I_ (mM)^a^		
	hCA I^b^	hCA II^b^	BloCAβ
F^‐^	> 300	> 300	> 100
Cl^‐^	6	200	> 100
Br^−^	4	63	92.8
I^−^	0.3	26	> 100
OCN^−^	0.0007	0.03	70.4
SCN^−^	0.2	1.6	15.8
CN^−^	0.0005	0.02	7.7
N_3_^−^	0.0012	1.5	0.9
HCO_3_^−^	12	85	84.8
CO_3_^2−^	15	73	75.9
NO_3_^−^	7	35	> 100
NO_2_^−^	8.4	63	> 100
HS^−^	0.0006	0.04	0.6
HSO_3_^−^	18	89	67.0
SO_4_^2−^	63	> 200	88.1
SnO_3_^2−^	0.57	0.83	25.1
SeO_4_^2−^	118	112	> 100
TeO_4_^2−^	0.66	0.92	> 100
P_2_O_7_^4−^	25.8	48	9.6
V_2_O_7_^4−^	0.54	0.57	0.4
B_4_O_7_^2−^	0.64	0.95	14.3
ReO_4_^−^	0.11	0.75	12.8
RuO_4_^−^	0.101	0.69	8.2
S_2_O_8_^2−^	0.107	0.084	1.7
SeCN^−^	0.085	0.086	70.6
CS_3_^2−^	0.0087	0.0088	0.3
Et_2_NCS_2_^−^	0.00079	3.1	0.09
ClO_4_^−^	> 200	> 200	9.5
BF_4_^−^	> 200	> 200	> 100
FSO_3_^−^	0.79	0.46	53.1
PF_6_^−^	> 50	> 50	> 100
CF_3_SO_3_^−^	> 50	> 50	3.0
NH(SO_3_)_2_^2‐^	0.31	0.76	30.9
NH_2_SO_2_NH_2_	0.31	1.13	4.9
NH_2_SO_3_H	0.021	0.39	0.8
Ph‐B(OH)_2_	58.6	23.1	5.5
Ph‐AsO_3_H_2_	31.7	49.2	> 100

^a^ Mean of three independent determinations performed using the stopped‐flow CO_2_ hydration assay; experimental errors were within ±5–10% of the reported values.

^b^ Data for hCA I and hCA II were taken from Bonardi et al. [37].

Overall, inhibitors **1**–**24** and AAZ‐HCT displayed moderate to weak inhibition of the bacterial isoform BloCAβ, showcasing *K*_I_ values in the range of 89.4 nM–39.3 µM. The strongest inhibition was observed for EZA (*K*_I_ = 89.4 nM), and BZA (*K*_I_ = 121 nM), which were approximately 5‐ and 3‐fold more potent than AAZ (*K*_I_ = 435 nM), respectively. Notably, **MZA** was also demonstrated to be twice as effective potent as AAZ, with a *K*_I_ of 281 nM. Moreover, DZA, BRZ, TPM, and IND displayed submicromolar *K*_I_ values ranging from 485 to 917 nM. Similarly, in the series **1**–**24**, compounds **14** and **19**–**24** exhibited submicromolar inhibition of the BloCAβ, with *K*_I_ values comparable to or lower than the clinical reference AAZ (*K*_I_ of 435 nM), highlighting their potential as leads; such *K*_I_s spanned between 412 nM and 883 nM. In particular, compound **14** showed the highest potency, *K*_I_ of 412 nM, followed closely by compounds **19** *K*_I_ of 461 nM, **20**, *K*_I_ of 543 nM, and **21**–**24** *K*_I_s ranging from 662 to 883 nM. All these inhibitors maintain good affinity across the three isoforms but show improved selectivity for hCA II. The remaining derivatives exhibited a poor inhibition of BloCAβ, with *K*_I_ values in the low‐to‐medium micromolar range, especially **SAC**, which showcased the worst activity, *K*_I_ of 39,300 nM. Similarly, derivatives **9**–**12**, SLP, and HCT demonstrated a comparable inhibition, with *K*_I_s ranging from 12.9 µM to 24.0 µM. Unfortunately, no compound outperformed its activity against hCA II, which is still the most inhibited isozyme, but a few derivatives, such as **1**, **2**, **4**–**8**, **13**–**17**, DZA, BRZ, VLX, and CLX, showed good selectivity for the bacterial BloCAβ instead of the cytosolic hCA I. In particular, VLX and CLX were approximately 21‐ and 25‐fold more potent against BloCAβ than against hCA I, respectively.

Anions and other small inorganic/organic compounds constitute a significant category of carbonic anhydrase inhibitors that primarily act as zinc‐coordinating ligands that bind to the metal ion within the enzyme's active site. In most CA classes containing zinc ions, these anions function as monodentate ligands, stabilizing the tetrahedral geometry of the metal centre. While trigonal bipyramidal coordination is occasionally observed, it remains rare. Due to their biological and pharmacological significance, this study evaluated a broad spectrum of organic and inorganic anions, as well as structurally analogous small molecules, all known to engage with metal ions in metalloenzyme active sites (Table 4) [56]. *K*_I_ values against BloCAβ are shown in Table 4. hCAs I and II inhibition data are included for comparison. The following should be noted regarding the data presented.

A group of inhibitors, including halides (except for Br^−^ exhibited a *K*_I_ value of 92.8 mM), NO_3_^−^, NO_2_^−^, SeO_4_^2−^, TeO_4_^2−^, BF_4_^−^, PF_6_^−^ and phenylarsonic acid (Ph‐AsO_3_H_2_) did not significantly inhibit BloCAβ up to 100 mM concentration in the assay system, although most of these show inhibition constants in the low‐ to sub‐millimolar range against hCAs I and II.

Several anions showed low to moderate inhibitory activity against BloCAβ (*K*_I_s ranging from 12.8 to 92.8 mM), indicating partial but significant interaction with the bacterial enzyme. This subset includes Br^−^, OCN^−^, SCN^−^, HCO_3_^−^, CO_3_^2−^, HSO_3_^−^, SO_4_^2−^, SnO_3_^2−^, B_4_O_7_^2−^, ReO_4_^−^, SeCN^−^, FSO_3_^−^ and NH(SO_3_)_2_^2−^. Within this range, ReO_4_^−^, B_4_O_7_^2−^, and SCN^−^ stand out as the most potent, although up to two orders of magnitude less selective than hCA I and hCA II. Several of these anions, such as HCO_3_^−^, CO_3_^2−^, HSO_3_^−^, and SO_4_^2−^, showed similarly weak inhibition across CA classes, indicating non‐selective interactions, possibly mediated by conserved electrostatic features along the enzyme active sites. Overall, this mid‐potency range captures a transitional class of inhibitors that do not fully exploit the binding features of BloCAβ, but which may serve as adaptable scaffolds for future inhibitor optimization toward bacterial CA selectivity. Remarkable inhibition of BloCAβ was observed for a smaller set of anions comprising CN^−^, P_2_O_7_^4−^, RuO_4_^−^, S_2_O_8_^2−^, ClO_4_^−^, CF_3_SO_3_^−^, NH_2_SO_2_NH_2_, and phenylboronic acid (Ph‐B(OH)_2_). The inhibitors showed *K*_I_ values ranging between 1.7 mM and 9.6 mM, suggesting more substantial engagement with the enzyme's active site. Notably, some anions in this subset exhibited higher selectivity for BloCAβ than for hCAs; for instance, triflate was roughly 15‐fold more potent against the bacterial enzyme than either human isozymes.

Sub‐millimolar potency was instead observed for N_3_^−^, HS^−^, V_2_O_7_^4−^, CS_3_^2−^, Et_2_NCS_2_^−^, and NH_2_SO_3_H, likely achieving tight binding through coordination with the active site Zn(II) ion and optimal positioning in the hydrophobic channel. Hence, despite their poor selectivity across CA classes, these anions represent the most promising chemical motifs for inhibitor design, particularly when subtle modifications could reduce hCA affinity, maintaining potency against the bacterial isoform. The distinct inhibition profiles presented here for bacterial and human CAs in response to various inorganic and organic anions highlight structural features that could be exploited to design selective inhibitors targeting bacterial enzymes without perturbing host physiological functions.

#### 2.4.2 Activation Profile of BloCAβ

**Figure 3:**
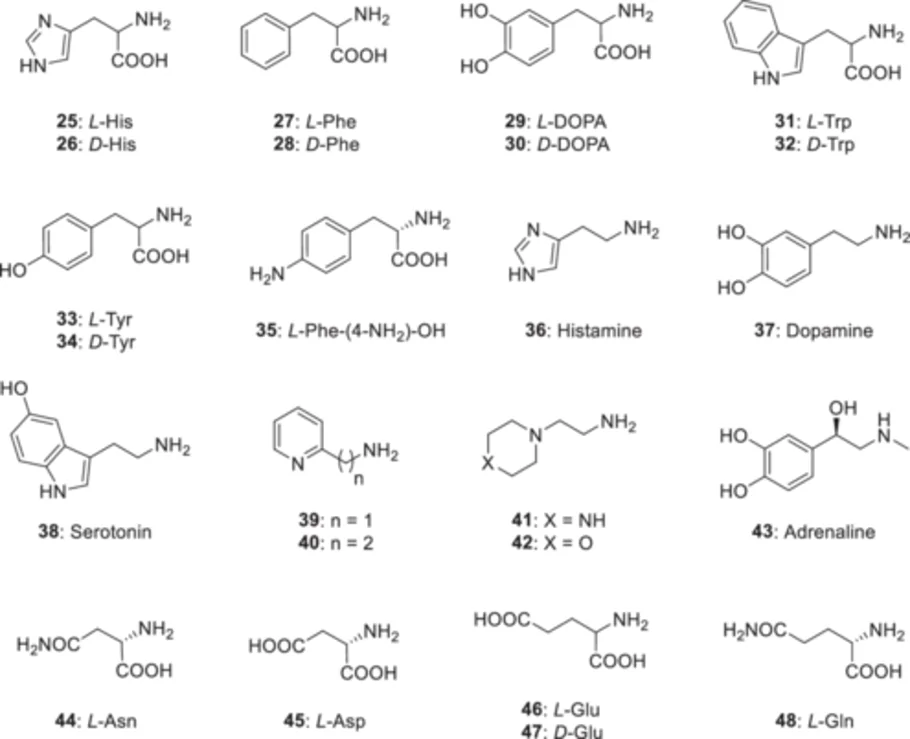
Chemical structures of the compounds used to evaluate the activation profile of BloCAβ. The tested panel comprised 24 compounds, including 16 amino acids and amino‐acid derivatives (**25–35** and **44–48**) and 8 amines (**36–43**).

The study of the activation profile of BloCAβ may provide useful information for future investigations aimed at understanding the physiological role of CA activity in probiotic bacteria. Although modulation of BloCAβ activity could potentially influence bacterial physiology, the present study was not designed to directly assess bacterial fitness, colonization capacity, competitive growth, or probiotic performance in vivo. Therefore, the identification of BloCAβ activators should be regarded as a biochemical starting point for future studies rather than as direct evidence of enhanced probiotic functionality. Thus, we further investigated activators **25–48** (Figure 3) belonging to the amino acid and amine chemotypesto assess their ability to activate BloCAβ and explore structure‐activity relationship.

In Table 5, the activation constants (*K*_A_) of compounds (**25–43**) against the target enzyme BloCAβ, as well as hCAs I and II, are shown for comparison. Among various activators for BloCAβ, the most potent were *L*‐Tyr (**33**), *K*_A_ of 0.65 µM, histamine (**36**), *K*_A_ of 0.59 µM, and *L*‐His (**25**), *K*_A_ of 0.84 µM. In particular, *L*‐Tyr (**33**) was approximately 5‐fold more potent than its enantiomer *D*‐Tyr (**34**), with a *K*_A_ of 3.4 µM. However, the *K*_A_ values for another enantiomeric pair, such as *L‐*His (**25**) vs *D*‐His (**26**), with *K*_A_s of 0.84 µM versus 1.7 µM, suggest that BloCAβ is modestly stereoselective, but both enantiomers often remain within single‐digit micromolar potency. This contrasts with hCAs I and II, where enantiomers can differ by orders of magnitude. In contrast, no enantiomeric selectivity was detected for *L*‐Phe (**27**) and *D*‐Phe (**28**), showcasing *K*_A_s in the low micromolar range, 7.0 and 6.2 µM, respectively. Interestingly, bulky or conformationally restricted aromatic amino acids, such as *L*‐Trp (**31**) and *D*‐Trp (**32**), were poor activators, displaying *K*_A_s in the medium micromolar range, 74.3 and 51.7 µM, respectively. This may indicate that large indole groups are detrimental, likely due to steric hindrance or reduced fit within the active site. Furthermore, catecholamines exhibited moderate to weak activity. Indeed, compounds such as dopamine (**37**), *K*_A_ of 39.8 µM, *L*‐DOPA (**29**), *K*_A_ of 47.5 µM, and l‐adrenaline (**43**), *K*_A_ of 52.7 µM, showed weaker activation, suggesting that the catechol motif, although effective in hCA II, is not favourably accommodated in the bacterial isoform. Moreover, simple amines, such as **39**–**42**, showcased weak potency against the bacterial isozyme, with *K*_A_ values ranging from 18.0 to 63.4 µM. Finally, *L*‐Asn (**44**), *L*‐Asp (**45**), *L*‐Glu (**46**), *D*‐Glu (**47**), and *L*‐Gln (**48**) all have *K*_A_s > 100 µM, suggesting that these polar side‐chain functionalities may reduce binding affinity.

**Table 5:** Activation of BloCAβ and hCAs I and II by amino acids, amino‐acid derivatives, and amines. *K*_A_ values were determined for compounds **25**–**48** using a stopped‐flow CO_2_ hydration assay.

Compound		*K*_A_ (µM)^a^		
		hCA I^b^	hCA II^b^	BloCAβ
**25**	*L*‐His	0.03	10.9	0.84
**26**	*D*‐His	0.09	43	1.7
**27**	*L*‐Phe	0.07	0.013	7.0
**28**	*D*‐Phe	86	0.035	6.2
**29**	*L*‐DOPA	3.1	11.4	47.5
**30**	*D*‐DOPA	4.9	7.8	22.4
**31**	*L*‐Trp	44	27	74.3
**32**	*D*‐Trp	41	12	51.7
**33**	*L*‐Tyr	0.02	0.011	0.65
**34**	*D*‐Tyr	0.04	0.013	3.4
**35**	4‐H_2_N‐*L*‐Phe	0.24	0.15	0.76
**36**	Histamine	2.1	125	0.59
**37**	Dopamine	13.5	9.2	39.8
**38**	Serotonin	45	50	26.4
**39**	2‐Pyridyl‐methylamine	26	34	57.2
**40**	2‐(2‐Aminoethyl)pyridine	13	15	48.6
**41**	1‐(2‐Aminoethyl)‐piperazine	7.4	2.3	18.0
**42**	4‐(2‐Aminoethyl)‐morpholine	0.19	0.94	63.4
**43**	*L*‐Adrenaline	0.09	96	52.7
**44**	*L*‐Asn	11.3	> 100	> 100
**45**	*L*‐Asp	5.2	> 100	> 100
**46**	*L*‐Glu	6.43	> 100	> 100
**47**	*D*‐Glu	10.7	> 100	> 100
**48**	*L*‐Gln	> 100	> 50	> 100

^a^ Mean of three independent determinations; experimental errors were within ±5%–10% of the reported values.

^b^ Data for hCA I and hCA II were taken from Bonardi et al., 2025 [37].

Given the valuable biological activity of *L*‐His (**25**), *L*‐Tyr (**33**), and histamine (**36**), and their therapeutic potential in promoting the activation of a probiotic bacterium, Table 6 presents a comparison of *K*_A_ values against BloCAβ and other β‐CAs from pathogenic microorganisms, such as *Burkholderia pseudomallei* (BpsCAβ), *Vibrio cholerae* (VchCAβ), *Escherichia coli* (EcoCAβ), *Mammalicoccus sciurii* (MscCAβ), *Mycobacterium tuberculosis* (MtbCA3β), *Brucella suis* (BsuCA1β), and *Francisella tularensis* (FtuCAβ), intending to highlight potential selectivity on the probiotic enzyme [36, 57, 58, 59].

**Table 6:** Activation of β‐CAs from different pathogenic microorganisms by *L*‐His (**25**), *L*‐Tyr (**33**), and histamine (**36**). *K*_A_ values were determined using a stopped‐flow CO_2_ hydration assay.

Compound		*K*_A_ (µM)^a^							
		BloCAβ	BpsCAβ^b^	VchCAβ^b^	EcoCAβ^b^	MscCAβ^b^	MtbCA3β^b^	BsuCA1β^b^	FtuCAβ^b^
**25**	*L*‐His	0.84	31.6	20.3	36.0	5.24	18.2	1.76	40.7
**33**	*L*‐Tyr	0.65	0.003	8.4	9.86	3.81	30.0	1.38	> 100
**36**	Histamine	0.59	0.012	9.5	18.5	1.15	34.2	3.71	> 100

^a^ Mean of three independent determinations performed using the stopped‐flow CO_2_ hydration assay; experimental errors were within ±5–10% of the reported values.

^b^ Data taken from Angeli et al. [36, 57, 58, 59].

As shown in Table 6, *l* ‐His (**25**) exhibited the most potent activation against BloCAβ, with a *K*_A_ in the submicromolar range, while only *K*_A_ values in the low‐to‐medium micromolar ranges were detected against all the other β‐CAs, ranging from 1.76 to 40.7 µM. In particular, Table 6 indicates a valuable selectivity for BloCAβ over the other β‐CAs. Conversely, *L*‐Tyr (**33**) and histamine (**36**) were slightly more potent BloCAβ activators than *L*‐His (**25**), but they also potently activated BpsCAβ, being approximately 217‐ and 49‐fold more potent against BpsCAβ than BloCAβ, respectively. However, a valuable selectivity was observed for all the other β‐CAs, which showed *K*_A_ values in the low‐to‐medium micromolar range, ranging from 1.15 to 34.2 µM. From a therapeutic perspective, the observed differences between the probiotic β‐class enzyme BloCAβ and the human α‐class CAs support the possibility of achieving selective enzyme modulation. The majority of clinically used sulfonamide CA inhibitors showed markedly greater potency against hCA II than against BloCAβ, while some compounds displayed preferential activity toward the bacterial enzyme relative to hCA I. This selectivity profile is encouraging for future microbiome‐directed therapeutic strategies, as it suggests that modulation of BloCAβ could potentially be achieved with limited off‐target effects on human CA isoforms.

### 2.5 Effects of Selected CA Activators on Human Macrophages and Human Intestinal Epithelial Cells

**Figure 4:**
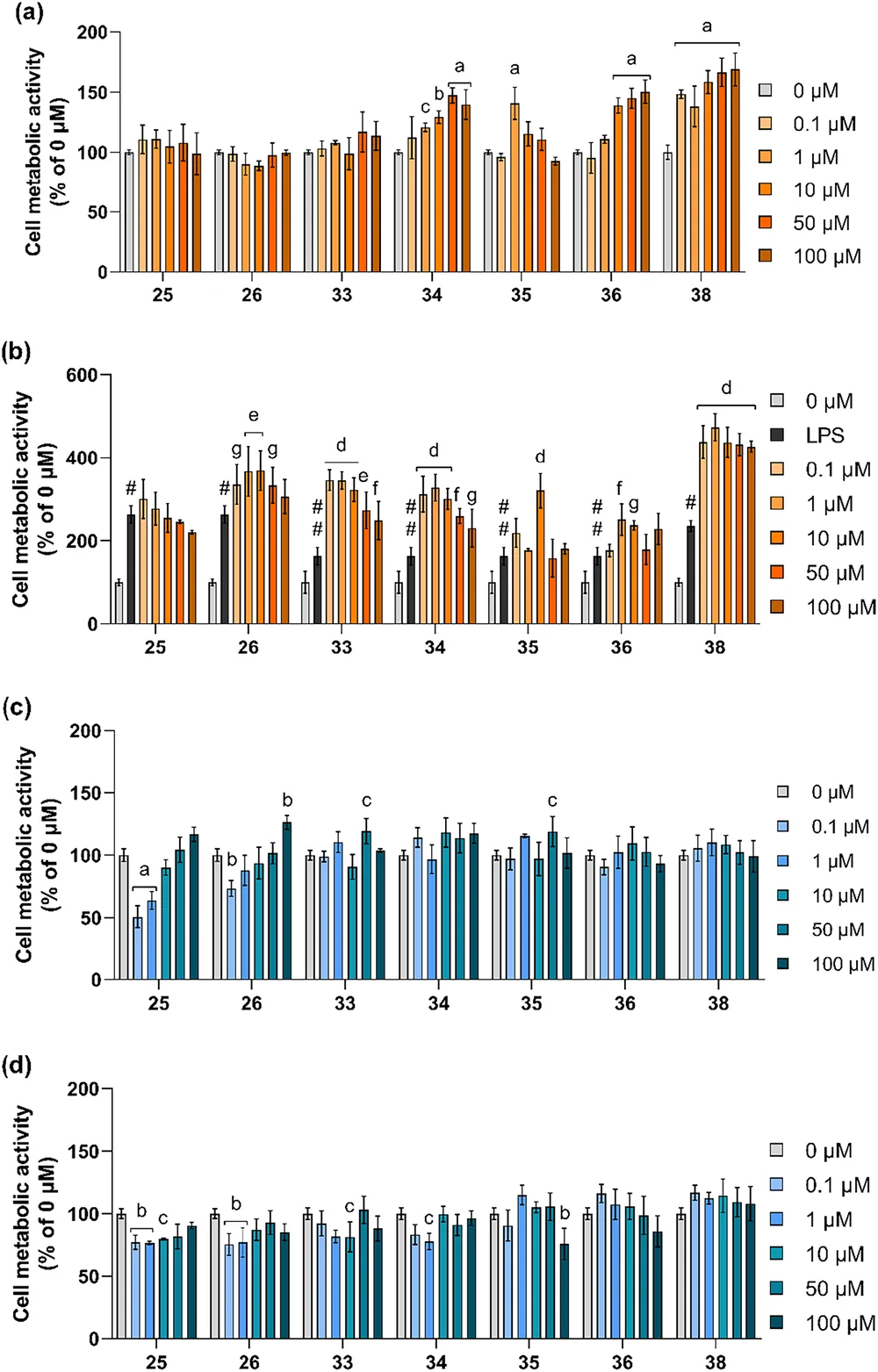
Effects of selected CA activators on cell metabolic activity. (a–b) Human macrophages were exposed to the indicated compounds for 24 h under (a) unstimulated or (b) lipopolysaccharide (LPS)‐stimulated conditions. (c–d) Human intestinal epithelial cells were treated with the same compounds for (c) 24 h or (d) 48 h. Untreated cells (0 µM) were used as the control, and their metabolic activity was set at 100%. Data are expressed as mean ± standard deviation (SD). Statistical significance versus untreated cells is indicated as follows: a *p* < 0.0001; b *p* < 0.0086; and c *p* < 0.0472. Statistical significance versus cells treated with LPS alone is indicated as follows: d *p* < 0.0001; e *p* < 0.0007; f *p* < 0.0067; and g *p* < 0.0491. Statistical significance of LPS‐treated cells versus untreated cells is indicated by #, *p* < 0.0001, and ##, *p* < 0.0010.

It should be emphasized that these cell‐based assays were designed to evaluate the biological compatibility and cellular effects of selected BloCAβ activators. Therefore, the observed changes in metabolic activity cannot be interpreted as direct evidence that activation of BloCAβ improves the fitness, colonization capacity, stress resistance, or probiotic functionality of *B. longum*. Dedicated studies employing live bacterial cultures and host–microbe interaction models will be necessary to address these aspects. The biological effect of selected CA activators was therefore evaluated on human macrophages (under basal and pro‐inflammatory conditions) after 24 h of exposure, and on human intestinal epithelial cells (HIEC‐6) after 24–48 h. The panel of activators (**25–26**, **33–36**, and **38**) was selected based on potency, stereochemistry, and isoforms selectivity, also considering their reported activity on another probiotic (*L. reuteri*) [37]. Under basal conditions (Figure 4a), the cell metabolic activity of macrophages is not significantly modulated except for *D*‐Tyr (**34**) and histamine (**36**), which stimulate the metabolism of macrophages in a dose‐dependent manner.

The stimulation with lipopolysaccharide (LPS) alone noticeably enhances the cell metabolic activity of macrophages, as a sign of immunostimulation [60]. It has been widely demonstrated that LPS induces oxidative stress in macrophages both in vitro and in vivo, and that stimulation with LPS is associated with the expression of oxidative stress regulators, inflammatory markers and related target genes [61]. None of the compounds fully counters the LPS‐induced increase in cell metabolic activity, (Figure 4b), although *L*‐His (**25**) shows a mild dose‐dependent decrease. Finally, serotonin (**38**) significantly increases cell metabolic activity in both basal and LPS‐stimulated conditions. In parallel, cell metabolic activity of HIECs is not significantly modulated (Figure 4c,d), except for *L*‐His (**25**) and *D*‐His (**26**), which lower the percentage of metabolically active intestinal cells in a dose‐dependent manner, thus highlighting the biocompatibility of these compounds.

## 3 Conclusions

In the search for probiotic CAs with functional relevance, we identified and annotated a novel β‐class isoenzyme from *B. longum*, named BloCAβ, through sequence alignment and phylogenetic analysis. This enzyme was successfully expressed, purified, and biochemically characterized. Notably, it was found to exhibit a catalytic efficiency within the same order of magnitude as, although lower than, that of hCA I.

Screening campaigns of sulfonamide‐based CA inhibitors revealed moderate‐to‐weak BloCAβ inhibition, with clinically used EZA and BZA emerging as the most potent. However, comparison of the inhibition profile of BloCAβ with those of hCAs I and II showed that no compound exceeded its potency toward hCA II, and several inhibitors displayed increased selectivity for BloCAβ over hCA I. A broad panel of inorganic and organic anions further highlighted distinct inhibition behaviors, identifying subsets of compounds with preferential affinity for the probiotic isoform. Furthermore, activation studies uncovered three potent BloCAβ activators, *L*‐His (**25**), *L*‐Tyr (**33**), and histamine (**36**), displaying submicromolar K_A_ values and modest stereoselectivity. Comparison with activation profiles of other pathogenic β‐CAs allowed the identification of *L*‐His (**25**) as the most selective BloCAβ activator, supporting its potential use as a selective chemical tool for investigating BloCAβ function.

Finally, selected CA activators underwent functional cellular assays. Most compounds, except for *D*‐Tyr (**34**) and histamine (**36**), had minimal effects on macrophage metabolic activity under basal conditions, whereas *L*‐His (**25**) showed modest, dose‐dependent activity in an LPS‐induced proinflammatory cell model. Serotonin (**38**) enhanced macrophage metabolic activity under both basal and inflammatory conditions, while maintaining biocompatibility in HIEC cells. Overall, these findings identify BloCAβ as a novel and biochemically tractable CA from *B. longum* and provide the first detailed characterization of its inhibition and activation profiles. Given the promising inhibitory and activating profiles observed for several compounds, these findings open the way to more in‐depth investigations aimed at clarifying the physiological role of BloCAβ and determining whether its pharmacological modulation may influence bacterial fitness, metabolic activity, colonization capacity, stress resistance, host–microbe interactions, and, ultimately, the probiotic functionality of *B. longum*.

The present results, therefore, support the further investigation of BloCAβ as a potential target for microbiome‐focused modulation strategies and provide useful chemical tools for exploring its biological and functional relevance. However, the current study does not demonstrate that activation of BloCAβ directly enhances probiotic fitness, bacterial colonization, stress resistance, host–microbe interactions, or therapeutic efficacy. These aspects require dedicated investigations in bacterial systems and appropriate gastrointestinal and in vivo models.

## 4 Material and Methods

### 4.1 Recombinant Production, Protein Isolation, and Characterization

#### 4.1.1 Chemicals and Instruments

Isopropyl β‐D‐1‐thiogalactopyranoside (IPTG) and antibiotics were purchased from Merck (Milan, Italy). His‐Trap FF affinity column and molecular weight markers were sourced from Cytiva (Uppsala, Sweden). Additional equipment included the AKTA Prime purification system (Cytiva), SX20 Stopped‐Flow instrument (Applied Photophysics, Leatherhead, UK), iBright 1500 Imaging System (Thermo Scientific, Waltham, MA, USA), and SDS‐PAGE apparatus (Bio‐Rad, Hercules, CA, USA). Unless otherwise stated, all other reagents were of analytical grade.

#### 4.1.2 Gene Identification, Synthesis, Cloning, and Heterologous Expression

Recombinant BloCAβ protein was produced through a combination of bioinformatics and molecular biology procedures. Initially, the *BloCAβ* gene was identified using the Protein BLAST program, performing various bacterial β‐CA sequences as queries. The gene encoding BloCAβ was designed and synthesized by a specialized company, GeneArt (Life Technologies, Milan, Italy), before being cloned into the pET100/d‐TOPO expression vector (Life Technologies, Milan, Italy). Then, the recombinant BloCAβ protein was expressed in *Escherichia coli* ArcticExpress competent cells and purified using a nickel‐based His‐Trap FF affinity column. Protein concentration was assessed via Bradford assay, whereas purity was evaluated by 12% SDS‐PAGE followed by Coomassie Brilliant Blue‐R staining. The final protein yield was 91%. Enzymatic activity was detected using a CA activity assay, which followed pH changes during the hydration of CO_2_ to bicarbonate at 0°C, and was quantified in Wilbur–Anderson units.

### 4.2 Enzymatic Activity Assessment

#### 4.2.1 BloCAβ Activity and Inhibition/Activation Measurements

An Applied Photophysics stopped‐flow instrument was used to assay the CA‐catalyzed CO_2_ hydration activity as previously reported [61]. Inhibition constants (*K*_I_) were obtained by non‐linear least‐squares methods using PRISM 7 and the Cheng–Prusoff equation from at least three different determinations. The activation constant (*K*_A_) was obtained by considering the classical Michaelis–Menten equation. The β‐CA from *B. longum* (BloCAβ) was recombinant and obtained *in‐house*. All tested compounds were purchased from Merck (Milan, Italy) and were of the highest purity available.

### 4.3 Cell‐Based Assays

#### 4.3.1 Human Cell Cultures

Undifferentiated human monocytes (CRL‐3622) were purchased from ATCC and cultured in complete RPMI 1640 (Merck, Darmstadt, Germany) at 37°C and 5% CO_2_, whereas human intestinal epithelial cells HIEC‐6 (CRL‐3266) were purchased from ATCC and cultured in complete DMEM/F12 (Merck, Darmstadt, Germany) supplemented with 4% inactivated fetal bovine serum (FBS), 1% penicillin/streptomycin, 20 mM HEPES, 10 mM glutamine (all from EuroClone, Milan, Italy) and 10 ng/mL of epidermal growth factor (EGF) (ThermoFisher Scientific, MA, USA) at 37°C and 5% CO_2_. Further details and monocyte differentiation into macrophages were described in our previous work [60].

#### 4.3.2 Human Cell Treatment and Establishment of Pro‐Inflammatory Conditions

Monocytes were seeded and differentiated as described previously [60]. The differentiation medium was discarded after 48 h and replaced with a fresh one containing complete RPMI (untreated cells) or compounds (**25**, **26**, **33–36**, and **38**) at 0.1, 1, 10, 50, and 100 µM (stock solutions: 100 mM in acetic acid 1% for compounds **25** and **26**; 100 mM in NaOH 1 N for compounds **33** and **34**; 100 mM in DMSO for compounds **35**, **36**, and **38**). To establish an inflamed environment, differentiated macrophages were stimulated with 0.5 µg/mL of lipopolysaccharide (LPS) from *E. coli* (Merck, Darmstadt, Germany) (stock solution 1 mg/mL in water) and exposed to compounds in parallel.

HIEC‐6 cells were seeded in 96‐well tissue culture‐treated plates at a density of 5 × 10^3^ cells/well. After 24 h, the medium was discarded and replaced with a fresh one containing complete DMEM/F12 (untreated cells) or compounds, as described above for differentiated macrophages.

#### 4.3.3 Human Cell Metabolic Activity

After 24 h from the treatment, the exposure medium of differentiated macrophages was replaced by 100 µL/well of fresh RPMI containing 0.5 mg/mL of 3‐(4,5‐dimethylthiazol‐2‐yl)−2,5‐diphenyltetrazolium bromide (MTT) (Merck, Darmstadt, Germany). Afterwards, cells were incubated for 4 h at 37°C. Then, the MTT medium was discarded, and 100 µL of DMSO were added to each well. In the end, samples were incubated for 10 min at 37°C. After 24 h and 48 h, the exposure medium of HIEC‐6 cells was replaced by 100 µL/well of fresh DMEM/F12 containing 0.5 mg/mL of MTT solution, and the MTT assay was performed as described above. The optical density in each well was measured at 540 nm. Each experiment was performed twice in triplicate (*n* = 6).

### 4.4 Statistical Analysis

Statistics were performed using two‐way analysis of variance (ANOVA) followed by Dunnett and Tukey's multiple comparison tests by means of the Prism 8.0 software (GraphPad, San Diego, CA, USA). Results are presented as mean values ± standard deviations. Values of *p* ≤ 0.05 were considered statistically significant.

## Consent

The authors have nothing to report.

## Conflicts of Interest

The authors declare no conflicts of interest.

## Supporting information


Supporting File 1

Supporting File 2

## Data Availability

The data that support the findings of this study are available from the corresponding author upon reasonable request.
