# Supplementary material for: From Identification to Enzymatic Activity Profiling: Comprehensive Characterization of BloCAβ, a Probiotic β‐Carbonic Anhydrase From Bifidobacterium longum
Source: Arch Pharm (Weinheim). 2026 Sep 16;359(9):e70333. doi: 10.1002/ardp.70333 (PMC13579351; doi:10.1002/ardp.70333)
Supplement: Supplementary file 1 [file ARDP-359-e70333-s002.docx]

Supporting information

**From Identification to Enzymatic Activity Profiling: Comprehensive Characterization of BloCAβ, a Probiotic β-Carbonic Anhydrase from *Bifidobacterium longum***

Simone Giovannuzzi,^a,§^ Andrea Ammara,^a,§^ Simone Carradori,^b,^* Viviana De Luca,^c^ Marialucia Gallorini^b^, Damiano Iacovozzi^b^, Amelia Cataldi^b^, Ilaria D’Agostino,^d^ Davide Moi,^e^ Valentina Onnis,^e^ Claudiu T. Supuran,^a^ Clemente Capasso^c^

*^a^NEUROFARBA Department, Section of Pharmaceutical Science, University of Florence, Via Ugo Schiff 6, 50019 Sesto Fiorentino, Florence, Italy*

*^b^Department of Pharmacy, “G. d’Annunzio” University of Chieti-Pescara, via dei Vestini 31, 66100 Chieti, Italy*

*^c^Department of Biology, Agriculture and Food Sciences, CNR, Institute of Biosciences and Bioresources, Napoli, Italy*

*^d^Department of Pharmacy, University of Pisa, via Bonanno Pisano 6, 56126 Pisa, Italy*

*^e^Department of Life and Environmental Sciences, Unit of Pharmaceutical, Pharmacological and Nutraceutical Sciences, University of Cagliari, Cittadella Universitaria di Monserrato, I-09042 Monserrato, CA, Italy*

*Corresponding author: Prof. Simone Carradori, simone.carradori@unich.it

^§^These authors contributed equally to this work

**Table of contents**

**Figure S1**. Phylogenetic analysis of BloCAβ.	S3

**Figure S2**. SDS-PAGE analysis of recombinant BloCAβ.	S3

**Figure S1.** Phylogenetic analysis of BloCAβ. A circular phylogenetic tree was constructed from a Clustal Omega multiple sequence alignment, including representative β-CAs from bacteria, plants, algae, and yeasts. Bootstrap analysis was performed with 1,000 replicates to assess the robustness of the branching topology. Red dots mark the nodes with bootstrap values as indicated in the bootstrap legend. All sequence acronyms and their corresponding organisms are listed in **Table 1**.

**Figure S2.** SDS-PAGE analysis of recombinant BloCAβ purified by nickel-affinity chromatography. A major band corresponding to an apparent molecular mass of ~29 kDa demonstrates successful expression and purification of the recombinant BloCAβ, containing the *N*-terminal His-tag.
